# Chromosomal regions strongly associated with waist circumference and body mass index in metabolic syndrome in a family-based study

**DOI:** 10.1038/s41598-021-85741-1

**Published:** 2021-03-16

**Authors:** Maryam S. Daneshpour, Maryam Zarkesh, Sajedeh Masjoudi, Fereidoun Azizi, Mehdi Hedayati

**Affiliations:** 1 Cellular and Molecular Endocrine Research Center, Research Institute for Endocrine Sciences, Shahid Beheshti University of Medical Sciences, PO Box, 19195-4763 Tehran, Iran; 2 Endocrine Research Center, Research Institute for Endocrine Sciences, Shahid Beheshti University of Medical Sciences, Tehran, Iran

**Keywords:** Biochemistry, Genetics

## Abstract

Obesity is the most crucial phenotype in metabolic syndrome (MetS), and waist circumference (WC) and body mass index (BMI) are two common indexes to define obesity. It is an accepted fact that genetic and environmental interaction influence obesity and MetS. Microsatellites are a subcategory of tandem repeats with a length of 1 to 10 nucleotides. Tandem repeats make up repetitive genomic regions. Differences in the number of tandem repeats or their variation (alleles) result in microsatellite polymorphisms. Thus, we attempted to find microsatellite variation associated with WC and BMI in a family-based study. Twelve microsatellite markers were selected to investigate possible genes or chromosomal regions in 91 families with at least one affected MetS. The cut-off values for BMI and WC were considered 25 kg/m^2^ and 90 cm, respectively. In all members of the families, the strongest association was observed between the marker D11S1304 (allele 1) with both WC and BMI, independently, by the biallelic model in the family-based association test analysis (P < 0.05). Besides, when we compared high- and low-level groups in members with MetS, the markers D8S1743 and D11S1304 (allele 1) showed a strong association with WC (P = 0.0080) and BMI (P = 0.0074), respectively. When the simultaneous detection of the high WC and MetS status was used as a trait, the strongest association was observed with the marker D8S1743 (P = 0.0034). Moreover, when BMI with the high MetS status was used as a trait, the strongest association was observed with the marker D8S1743 (allele 4) (P = 0.0034). The obtained results showed a relationship between obesity and MetS with markers on the selected regions on chromosomes 8 and 11, and to a lesser degree, on chromosome 12.

## Introduction

As a multiple-component condition, metabolic syndrome (MetS) can be called a multiplex cardiovascular risk factor. Insulin resistance, glucose intolerance, hypertension, dyslipidemia, and obesity, in particular central obesity, are known as MetS^1,2^. The prevalence of obesity in Iranian men and women is 21% and 38.6%, respectively^4^. Thus, obesity is the most critical factor in the high prevalence of MetS in Iran^3^. It is accepted that this syndrome results from a complex interplay of genetic and environmental factors. Moreover, genetic variants underlying metabolic traits have been identified at several loci in Iranian populations^5–12^. Identifying new chromosomal loci through association studies can help discover new genes responsible for obesity.

The genome-wide analysis has recently identified genetic variants in association with waist circumference (WC) and BMI^13,14^. These studies found some chromosomal regions such as 8(q22.1-q24.3) containing *VPS13B* gene^15–22^, 11(q23.3-q25) containing *APOA5, ZPR1,* and *BUD13* genes^23–28^, 12(q13.12-q15) containing *FAIM2* gene^18,29–32^, and 16(q23.3-q24.3) containing *MAF* gene^31–33^ with high LOD scores concerning BMI and WC (Table 1). Even some genetic variations were reported concerning MetS in the Japanese population^34^. We attempted to evaluate the effect of variation in the selected regions on obesity in MetS.

**Table 1:** Essential genes in the selected chromosomal region.

Region	Closest gene(s)	Phenotype	Proposed molecular or cellular function	References
8q22.2	*VPS13B*	Abnormal fat storage, obesity, Cohen syndrome	Glycosylation flaws and dysfunction of Golgi	^21,22^
11q23.3	*APOA5*	BMI/metabolic traits	The increased risk of MetS and its components, especially elevated TG and low HDL-C levels	^27,28^
11q23.3	*ZPR1(ZNF259), BUD13*	Serum lipid level elevated TG level	Insulin sensitivity and obesity	^27^
12q13.12	*FAIM2*	BMI	Adipocyte apoptosis	^31,32^
16q23.2	*MAF*	BMI	Transcription factor involved in adipogenesis and insulin–glucagon regulation	^31,32^

Since obesity is an essential component in MetS in Iranian patients, this study aimed to replicate the linkage and association of some chromosomal regions previously reported to be involved in obesity-related factors such as WC and BMI in Iranian families with MetS.

## Results

### Characteristics of the study population

This study included a total of 490 members of 91 families. Of this number, 122 (24.4%) had MetS, 143 (28.1%) had WC ≥ 90 cm, and 138 (27.2%) suffered from BMI ≥ 25 kg/m^2^. The demographic and biochemical characteristics of all the studied subjects are presented in Table 2.

**Table 2:** The demographic and biochemical parameters of participants in the study.

Characteristic	Total
	N ^a^ = 508
Age (years)	36.6 ± 19.1 ^b^
Male/female (n)	247/261
Body Mass Index (BMI) (kg/m^2^)	26.1 ± 5.47
**Risk factors for the metabolic syndromes**	
**Waist circumference (Cm)**	
Women	84.2 ± 15.4
Men	90.4 ± 15.4
Fasting plasma glucose (mg/dl)	95.1 ± 27.3
**Elevated blood pressure (mmHg)**	
Systolic	114 ± 20.5
Diastolic	71.1 ± 10.8
Serum triglycerides (mg/dl)	143 ± 89.1
Cholesterol (mg/dl)	185 ± 40.8
**HDL cholesterol (mg/dl)**	
Women	47.5 ± 11.7
Men	40.6 ± 9.56

^a^The number of subjects in each group.

^b^Mean ± standard deviation.

### FBAT analysis

The biallelic model results in the family-based association test (FBAT) analysis for high and low WC and BMI levels in all the family members are provided in Table 3. The strongest association was independent with the marker D11S1304 (allele 1) for WC and BMI (Z = 2.124, P = 0.0337, and Z = 2.253, P = 0.0253, respectively). Although, after correcting the p-value for multiple testing (Bonferroni’s correction), this marker did not last as a significant marker (P = 0.069). Moreover, the multiallelic analysis did not reveal the association with this marker (P = 0.086).

**Table 3:** The biallelic model results in the FBAT analysis of high waist circumferences and high BMI.

Biallelic model							
Marker		High waist			High BMI		
Name	Allele ^a^	Frequency	Z ^b^	P	Frequency	Z	P
D8S1132	3	0.187	1.82	0.0687	–	–	–
D8S1132	18	0.066	–1.893	0.0583	–	–	–
D8S1743	4	0.331	1.739	0.0821	0.331	1.879	0.0602
D8S1743	5	–	–	–	0.09	− 2	**0.0455**
D8S1743	12	0.016	− 1.667	0.0956	–	–	–
D11S934	2	0.013	− 2.236	**0.0253**	0.013	− 2.236	**0.0253**
D11S1304	1	0.363	2.124	**0.0337**	0.363	2.253	**0.0243**
D11S1304	2	0.231	− 1.695	0.0901	–	–	–
D12S96	4	0.042	− 2.714	**0.0067**	0.042	− 1.941	0.0522
D12S329	2	0.078	− 2.711	**0.0067**	0.078	− 2.064	**0.0390**
D12S329	5	0.065	− 2	**0.0455**	–	–	–
D16S2624	1	–	–	–	0.185	− 1.729	0.0839
D16S2624	2	0.316	1.81	0.0704	–	–	–
D16S3096	15	–	–	–	0.016	− 1.89	0.0588

^a^Allele name according to the number of repeats.

^b^Value of the Z test of association.

The association results of WC, the BMI level, and the MetS status in the FBAT analysis are shown in Table 4.

**Table 4:** The association results of waist circumference, BMI level, and MetS status in the FBAT analysis.

Marker	Allele ^a^	Waist							BMI						
		(Low waist/high waist)/MetS status			MetS status/waist circumference				(Low BMI /high BMI)/MetS status			MetS status/BMI			
		Frequency	Z ^b^	P ^c^	Frequency	Z	P	Waist^d^	Frequency	Z	P	Frequency	Z	P	BMI
D8S1132	17	0.064	2.153	0.0313	0.064	2.251	0.0244	85.9 ± 15.6	0.064	2.243	0.0249	0.064	2.351	0.0528	25.8 ± 5.97
D8S1779	3	0.289	2.047	0.0407	–	–	–	–	0.289	2.174	0.0297	–	–	–	–
D8S1743	4	0.331	2.651	0.0080	0.331	2.931	**0.0034**	87.2 ± 15.2	0.331	2.599	0.0093	0.331	2.842	**0.0024**	25.9 ± 5.50
D8S1743	7	0.026	− 2.097	0.0360	–	–	–	–	0.026	− 2.140	0.0324	–	–	–	–
D8S1743	13	0.016	− 2.130	0.0331	0.016	− 2.165	0.0304	85.5 ± 14.7	0.016	− 2.100	0.0357	0.016	− 2.105	0.0450	26.3 ± 6.12
D11S1998	7	–	–	–	–	–	–	–	0.013	− 1.970	0.0490	–	–	–	–
D11S934	1	0.254	2.092	0.0365	0.254	1.963	0.0496	86.1 ± 15.8	–	–	–	0.254	1.998	0.0482	25.7 ± 5.53
D11S1304	1	0.363	2.583	0.0098	0.363	2.849	**0.0044**	87.9 ± 15.2	0.363	2.679	0.0074	0.363	2.640	**0.0025**	26.4 ± 5.53
D11S1304	6	0.023	− 2.934	**0.0034**	0.023	− 2.828	**0.0047**	87.8 ± 15.8	0.023	− 2.950	**0.0032**	0.023	− 2.750	**0.0032**	26.1 ± 5.21
D12S329	1	0.035	− 2.238	0.0252	0.035	− 1.995	0.0461	94.8 ± 14.1	0.035	− 2.330	0.0200	0.035	− 1.932	0.0532	28.7 ± 5.64
D16S2624	1	–	–	–	0.185	− 2.083	0.0372	86.9 ± 14.2	–	–	–	0.185	− 2.148	0.0408	25.7 ± 5.36
D16S2624	2	0.316	2.370	0.0178	0.316	2.395	0.0166	87.2 ± 15.1	0.316	2.404	0.0162	0.316	2.652	0.0252	25.9 ± 5.25

^a^Allele name according to the number of repeats.

^b^Value of the Z test of association.

^c^In bold, significant p-values after Bonferroni correction for multiple testing (global p < 0.05).

The strongest association was observed with the marker D8S1743 for high and low WC in individuals with MetS (Z = 2.651, P = 0.0080). When the simultaneous detection of the high WC and MetS status was investigated, the marker D8S1743 (Z = 2.931, P = 0.0034) had the strongest association. Furthermore, after correcting the p-value for multiple testing (Bonferroni’s correction), there were significant associations with the marker D11S1304 (allele 6) in high and low WC with the MetS status group (P = 0.0405) and with the markers D8S1743 (allele 4) (P = 0.0342), D11S1304 (allele 1) (P = 0.0432), and D11S1304 (allele 6) (P = 0.0351) in WC with the MetS status group.

Besides, the highest association was observed to be with the marker D11S1304 (allele 1) for high and low BMI in individuals with MetS (Z = 2.679, P = 0.0074). When the simultaneous occurrence of high BMI with the MetS status was used as a trait, the strongest association was observed with the marker D8S1743 (allele 4) (Z = 2.931, P = 0.0034). After correcting the p-value for multiple testing (Bonferroni’s correction), there were significant associations with the marker D11S1304 (allele 6) in high and low BMI with the MetS status group (P = 0.0351) and with the markers D8S1743 (allele 4) (P = 0.0254), D11S1304 (allele 1) (P = 0.362), and D11S1304 (allele 6) (P = 0.0381) in BMI with the MetS status group. These results suggested an association with regions on chromosomes 8 and 11, and to a lesser degree, on chromosome 12.

### Linkage analysis

**Figure 1:**
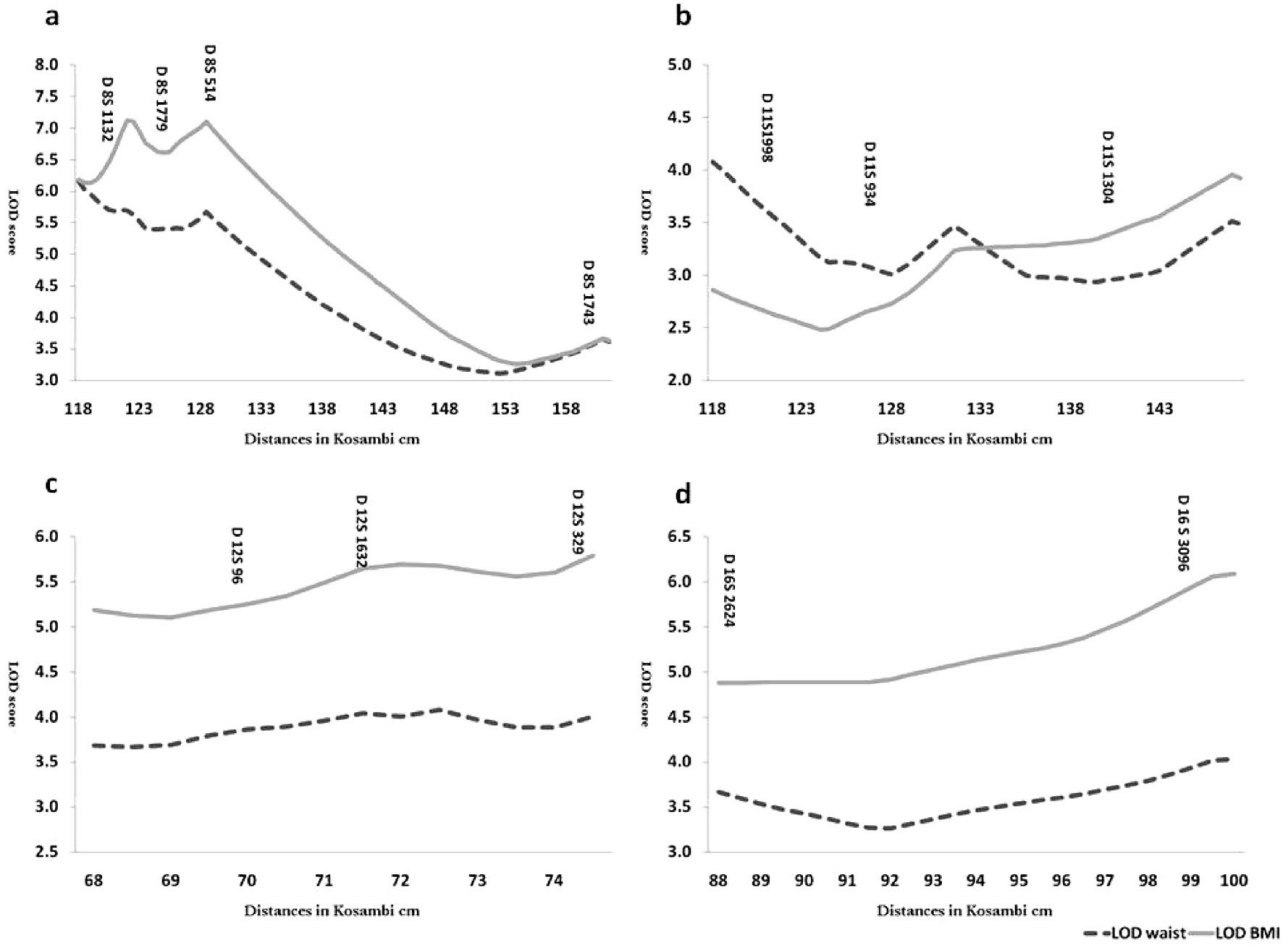
The pooled LOD score for families with the positive score: (**A**) chromosome 8, (**B**) chromosome 11, (**C**) chromosome 12, and (**D**) chromosome 16.

Nonparametric linkage analysis (NPL) performed by Merlin software yielded no significant result for any of the chromosomal regions. The highest NPL score (on the LOD scale) was 1.2 for WC and 1.62 for BMI for the marker D8S514. This result might be explained by the fact that many families are not informative for the studied markers (since the maximal attainable LOD was 1.55). However, if only families with positive nonparametric LOD scores and added LODs were considered, different results would be obtained (Fig. 1). In this figure, 37 families for Chr8, 36 families for Chr11, 31 families for Chr12, and 38 families for Chr16 contributed. LOD scores were above the significance threshold (> 3) for all the markers in all the regions.

A parametric analysis was performed under both dominant and recessive analyses (with 80% prevalence and 0.01 disease gene frequency). Although no significant LOD score was found in any of the chromosomal regions, very informative families for the linkage under both modes of inheritance were identified (Table 5). For example, the family 12,422 had the LOD score > 1 for the marker on chromosome 12 (LOD 1.12 for D12S329) based on WC and BMI as a trait. Similar patterns were observed for the family 30,250 with the same marker in groups with WC affection by the 1.01 LOD score. The family 21,030 was identified as being informative and had the linkage under both dominant and recessive models to regions on chromosomes 8 (LOD > 0.7) (Table 5). When the LOD scores of families with positive values were added together, we obtained a global LOD score of about 7.1 for 8q22-24. The pooled LOD score for positive families reached 2.5 for chromosome 11.

**Table 5:** The parametric linkage analysis results under both dominant and recessive analyses.

Region	Model	BMI				Waist			
		Family	Position	Microsatellite	LOD score	Family	Position	Microsatellite	LOD score
8q22-24	Dominant	21,030	128.9	D8S514	0.6964	10,608	128.9	D8S514	0.8597
	Recessive	–	–	–	–	21,030	128.9	D8S514	0.6615
	Dominant	12,622	128.9	D8S514	0.7067	20,868	128.9	D8S514	0.7268
	Dominant	21,030	122.6	D8S1779	0.8527	10,608	122.6	D8S1779	0.884
	Recessive	–	–	–	–	21,030	122.6	D8S1779	0.7124
	Dominant	31,563	161.5	D8S1743	0.6286	20,868	117.9	D8S1132	0.7618
	Dominant	21,030	117.9	D8S1132	0.7137	30,615	117.9	D8S1132	0.6642
11q23-25	Dominant	20,232	131.7	D11S934	0.874	20,232	131.7	D11S934	0.874
	Dominant	12,422	131.7	D11S934	0.8998	12,422	131.7	D11S934	0.8998
12q13-15	Dominant	21,464	67.16	D12S96	0.7709	21,464	67.16	D12S96	0.7709
	Dominant	31,682	74.58	D12S329	0.6042	31,682	74.58	D12S329	0.9192
	Dominant	10,501	74.58	D12S329	0.6258	11,983	67.16	D12S96	0.7424
	Dominant	12,422	74.58	D12S329	**1.1242**	12,422	74.58	D12S329	**1.1242**
	Dominant	–	–	–	–	30,250	74.58	D12S329	**1.0115**
	Dominant	–	–	–	–	22,305	74.58	D12S329	0.7287
	Dominant	–	–	–	–	11,983	71.61	D12S1632	0.7307
16q23-24	Dominant	20,232	97.3	D16S3096	0.9302	20,232	97.3	D16S3096	0.9302
	Dominant	–	–	–	–	31,682	97.3	D16S3096	0.9336
	Dominant	–	–	–	–	32,189	97.3	D16S3096	0.8304
	Dominant	31,682	86	D16S2624	0.6052	31,682	86	D16S2624	0.9338
	Dominant	33,372	86	D16S2624	0.6716	20,868	86	D16S2624	0.9906
	Dominant	–	–	–	–	32,189	86	D16S2624	0.7007

Bold families have high LOD scores in both traits.

## Discussion

Obesity is the most critical component of MetS in the Iranian population. Thus, four chromosomal regions in 91 families with MetS and high WC and BMI patterns were selected to study the genetic cause of these problems. The genetic transmissions of 12 microsatellite markers were studied, and association analysis was carried out. The FBAT generated nominal support for the allelic association in D8S1743 and D11S1304 in biallelic mode. The linkage analysis yielded no significant results for any of the chromosomal regions. Still, when we considered only families with positive nonparametric LOD scores and added their LODs, LOD scores were above the three thresholds for all the markers in all the regions. These results are in good agreement with the FBAT results since our results are in agreement with previous findings.

The biallelic FBAT analysis results showed that the 12q13-15 chromosomal region with the D12S329 microsatellite marker had a significant association with WC and BMI variation; however, this relation disappeared after Bonferroni correction. This marker has the highest LOD score in Iranian families under the dominant model, whereas the marker D8S1743 was the most significant under the recessive model and in association tests. Norris et al.^18^ found a high LOD score in this region for the waist to hip ratio. After using WC and BMI as a trait in the FBAT analysis, D11S1304 showed a strong relationship with all situations. It should be noted that these regions harbor the *ApoAI* gene cluster^25^. Palmer et al.^35^ used 59 large pedigrees to identify a locus on chromosome 8q21.3 for the body mass index (BMI) with markers D8S1179 and D8S1128 yielding the highest LOD scores (> 2) under a model-free linkage analysis. One of the studies' limitations is the low number of patients with MetS and without a high WC. These patients are rare per definition, but they exist, and of course, this would have been the best negative control group.

In summary, the obtained results suggest that regions 8q22-24, 11q23-25, and 12q13-15 are very likely to contain genes like *VPS13B*, *APOA5*, *ZPR1*, *BUD13*, and *FAIM2* that control obesity-related factors in Iranian families with MetS^21,22,27,28,31,32^. A more refined analysis with high-density short tandem repeat (STR) markers in the regions of interest around the marker with the most robust evidence for linkage and association, which possibly focuses on very informative families identified in this study, is needed to pave the way to identify responsible genes.

## Materials and methods

This study was conducted within the framework of the Tehran Lipid and Glucose Study (TLGS). The TLGS is a large-scale community-based prospective study performed on a representative sample of residents in district 13 of Tehran^36^. The subjects were selected from the third examination phase (2006–2008) of the TLGS. All the subjects answered a questionnaire on demographic and biochemical factors and smoking habits at study entry. Informed written consent was obtained from all the participants. The ethics committee approved this study in the Research Institute for Endocrine Sciences. All methods of the study were performed following the relevant guidelines and regulations.

### Phenotype measurement

The height, weight, WC, and blood pressure of each individual were measured, and BMI was calculated by dividing weight (kilogram) by the square of height (m^2^). Fasting blood glucose, total cholesterol, HDL-C, and triglyceride levels were measured immediately from fresh sera, as described previously^37,38^. Serum HDL-C levels were measured after the precipitation of ApoB containing lipoproteins with dextran-magnesium sulfate^39^. LDL-C and VLDL concentrations in samples with serum triglyceride levels < 400 mg/dl were calculated using Friedewald’s equation and one-fifth of triglyceride levels, respectively^40^. Coefficients of variation (CV) for total cholesterol, HDL-C, and triglyceride measurements were below 5%. Waist circumference ≥ 90 cm and a BMI ≥ of 25 kg/m^2^ were recorded as main phenotypes.

### Family selection

Families were selected based on having at least one member affected by MetS. MetS was defined based on the Third Report of the Expert Panel on Detection, Evaluation, and Treatment of High Blood Cholesterol in Adults (ATP III), which was modified locally^41^. Among them, families with at least two members with WC ≥ 90 cm and BMI ≥ 25 kg/m^2^ in a different generation were selected^41^. Families in this sample were 91 pedigrees, 201 nuclear families, 508 individuals, and three-generation families comprising 295 parents, 273 full siblings, and 11 grandparents.

### Marker selection

Regions with high LOD scores (> 3) were selected according to the previous studies, including 8(q22.1-q24.3)^15–20^, 11(q23.3-q25)^23–26^, 12(q13.12-q15)^18,29,30^, and 16(q23.3-q24.3)^33^. Among them, 12 highly informative STR loci were selected to allow good coverage of the regions: D8S1132, D8S1779, D8S514, D8S1743, D11S1304, D11S1998, D11S934, D12S1632, D12S329, D12S96, D16S3096, and D16S2624.

### Genotyping of microsatellite markers

Buffy coats were separated from the non-coagulated blood samples and stored at -70 °C until processing. Genomic DNA was extracted by the Proteinase K, salting out the standard method^42^. The Gene Amp PCR System 9700 (ABI Co. USA) was used to simultaneously amplify 12 STR loci, including D8S1132, D8S1779, D8S514, D8S1743, D11S1304, D11S1998, D11S934, D12S1632, D12S329, D12S96, D16S3096, and D16S2624. Amplification was performed, as previously described^43^. The raw data were analyzed using the ABI Data Collection Software and Gene Mapper 3.2 (Applied Biosystems). Laboratory internal control standards were used for quality control.

### Statistical analysis

The Cyrillic program drew family pedigrees, and PedCheck^44^ and Merlin^45^ checked Mendelian and possible genotyping errors. Markers with incompatible or unlikely genotypes were removed for the concerned individuals. Marker allele frequencies were estimated from the founders of the 91 families^38^. Marker map positions were based on the sex-average maps from the Marshfield Medical Research Foundation^16^. The FBAT package was used to test for association in the presence of linkage^46^. FBAT provided a Chi-square test that examined the composite null hypothesis of no linkage or no linkage disequilibrium, correcting for confounding due to admixture. Merlin software performed multipoint nonparametric linkage analysis^45^. Merlin outputs were nonparametric NPL_all_ (Z) and LOD scores and their corresponding asymptotic p-values. Corrected p-values based on Bonferroni’s correction (multiplying the uncorrected p-values by the number of alleles) were also reported for association tests.
